# Dyskeratosis Congenita: A Report of Two Cases

**DOI:** 10.1155/2013/845125

**Published:** 2013-08-06

**Authors:** Anila Karunakaran, Rathy Ravindran, Mohammed Arshad, M. Kodanda Ram, M. K. Shruthi Laxmi

**Affiliations:** ^1^Department of Oral Pathology and Microbiology, Kannur Dental College, Anjarakandy, P.O. Mamba, Kannur 670611, India; ^2^Department of Oral Pathology and Microbiology, Azeezia College of Dental Science & Research, Diamond Hills, Meeyannoor, Kollam 691537, India; ^3^Department of Preventive and Community Dentistry, Kannur Dental College, Anjarakandy, P.O. Mamba, Kannur 670611, India

## Abstract

Oral manifestations play an important role in the diagnosis of many systemic conditions. Dyskeratosis congenita (DC) is a rare genodermatosis which exhibits oral leukoplakia, nail dystrophy, and reticular skin pigmentations as its primary features. DC has increased risk of developing constitutional anemias and malignancies and early diagnosis enables the patient to be monitored and proper interventional therapy to be instituted. Hence, dentists need to be aware of the various manifestations of this fatal syndrome. Only few cases have been reported on DC in the dental literature. Two cases of DC are reported here with a brief review of the literature.

## 1. Introduction

Dyskeratosis congenita (DC) is a rare inherited bone marrow failure syndrome characterized by the triad of dystrophy of the nails (90%), reticular skin pigmentation (90%), and oral leukoplakia (80%). It is associated with a high risk of developing aplastic anemia, myelodysplastic syndrome, leukemia, and solid tumors. Atresia of the lacrimal ducts may occur causing continuous lacrimation. Patients have very short germline telomeres. Hence, many of the associated symptoms like premature graying are characteristic of geriatrics and the tissues affected are those with a high cell turnover [1].

In this paper two cases of  DC both showing oral mucosal changes are reported.

## 2. Case 1 (Figure 1)

A girl aged 11 reported with symptoms of burning sensation of the tongue since one year.

The patient showed pallor of the face but vital signs were within normal limits.

She was not exposed to tobacco in any form. There was no family history. Her milestones were normal up to 1 year after which they slowed down.

**Figure 1:**
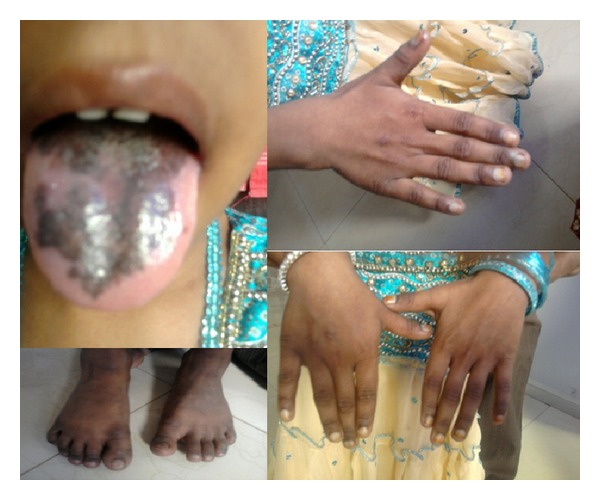
Case 1—clinical picture showing oral leukoplakia, nail dystrophy, and hyperkeratotic pigmented patches on hands and feet.

Intraorally, the tongue showed an extensive leukoplakia of size 5 × 7 cm with black pigmentation and well-defined borders (Figure 1). The pigmentation had appeared subsequent to the leukoplakia and slowly increased to the present intensity. No induration was noted.

There was mild dystrophy with splitting of the fingernails since 5 years (Figure 1). Hyperkeratotic and pigmented patches were present on the back, feet, and hands (Figure 1). No soreness or watering of the eyes was noted.

Blood examination showed Hb 6 gm%, prothrombin time 30 sec, and white blood cell count 6000 per cu mm, indicating a pancytopenia.

## 3. Case 2 (Figure 2)

A female patient aged 20 presented with complaint of a white patch on the tongue with burning sensation since six months. This was previously diagnosed as lichen planus and she was put under oral steroids. There was black pigmentation on the white patch which reportedly disappeared after the previous treatment.

**Figure 2:**
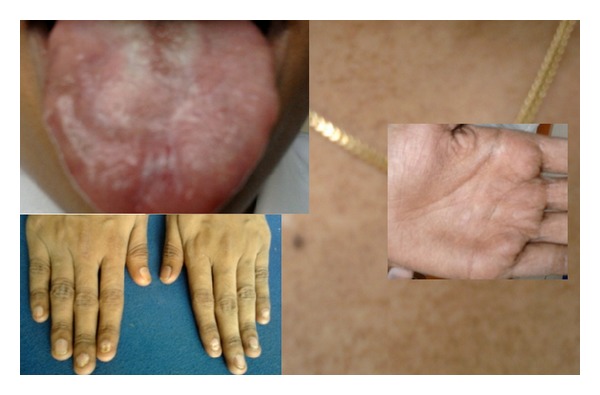
Case 2—clinical picture showing oral leukoplakia, nail dystrophy, and reticular pigmentation on neck and palm.

Intraoral examination revealed a bald tongue with a leukoplakic patch of size 3 × 4 cm. The depapillated regions were erythematous (Figure 2).

Patient did not have a history of tobacco usage. No family history was reported.

There was dryness of skin with reticular pigmentation on the sun exposed areas, especially the back and the neck, as well as the palms and soles since three years (Figure 2). She also had brittle and cracked nails which were painful and present for the same period (Figure 2). Occasionally, there was pus discharge from the nails which had been treated with antibiotics. There was associated sweating of palms and soles of feet. Patient had mild photophobia and epiphora. There was a significant alopecia of scalp over the past year.

Blood picture, however, was satisfactory.

## 4. Differential Diagnosis

Differential diagnosis of the previous cases included Fanconi's anemia, pachyonychia congenita, white spongy nevus, and Graft versus Host disease [2].

**Figure 3:**
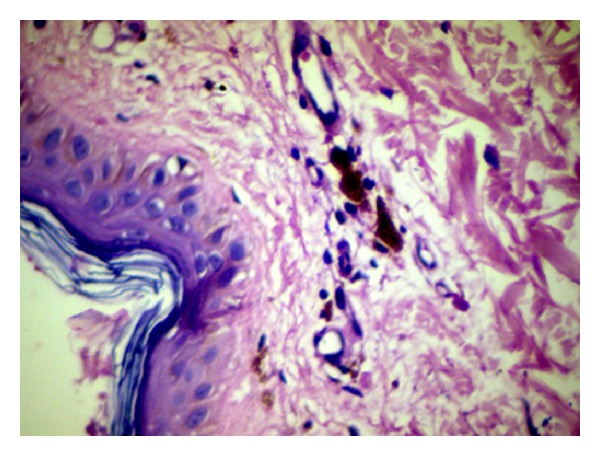
H&E section of the pigmented area of upper back of case 2 showing atrophic epithelium overlying a moderately collagenous connective tissue. Melanocytes present in the dermis. Inflammatory cells are conspicuous by their absence (high power).

Biopsy was taken from the skin of the back in both cases and diagnosis of DC was confirmed (Figure 3).

## 5. Discussion

DC was first described by Zinsser in 1910 and later by Engman and by Cole et al. leading to the designation of Zinsser-Engman-Cole syndrome [3]. Hyper- or hypopigmentation of tan-to-gray colour, in a mottled or reticulated pattern, presenting as macules and patches is the primary diagnostic feature. Poikilodermatous changes with atrophy and telangiectasia are common. The sun-exposed areas, including the upper trunk, neck, and face, are the most affected areas. Ectodermal abnormalities such as alopecia of the scalp, eyebrows, and eyelashes; premature graying of the hair; hyperhidrosis; hyperkeratosis of the palms and soles; and adermatoglyphia (loss of dermal ridges on fingers and toes) are noticed [4].

Approximately 90% of patients exhibit nail dystrophy. The fingernails are involved prior to toenails in most cases. Nail dystrophy begins with ridging and longitudinal splitting and progresses resulting in small, rudimentary, or absent nails [4].

Mucosal leukoplakia is a pathognomonic feature and occurs in approximately 80% of patients. It typically involves the buccal mucosa, tongue, and oropharynx [4]. Leukoplakic areas show an increased risk of malignant transformation and hence require frequent monitoring.

Approximately 90% have peripheral cytopenia of one or more lineages. In some cases, this is the initial presentation, with a median age of onset of  10 years. Adverse events include severe bone marrow failure, myelodysplastic syndrome, acute myeloid leukaemia, and solid tumours. Both Fanconi's anemia and DC are major cancer susceptibility syndromes [5, 6]. Bone marrow failure is a major cause of death, with approximately 70% of deaths related to bleeding and opportunistic infections occurring as a result [7].

Pulmonary complications, including pulmonary fibrosis and abnormalities of pulmonary vasculature, are seen in about 80% of cases [7].

Patients have an increased prevalence of malignant mucosal neoplasms, particularly squamous cell carcinoma. These often occur within sites of leukoplakia. The prevalence of squamous cell carcinoma of the skin is also increased. Other malignancies reported include Hodgkin's lymphoma, adenocarcinoma of the gastrointestinal tract, and bronchial and laryngeal carcinoma [2]. Malignancy tends to develop in the third decade of life [6].

The skeletal, gastrointestinal, and genitourinary systems also may be affected.

Female carriers of DC may have subtle clinical features.

To date, mutations in six genes of telomerase and telomere components have been identified in patients with DC [1]. Extreme telomere shortening causes the clinical features of DC. Alterations in posttranslational modification of ribosomal and spliceosomal RNAs may also play a role in the pathogenesis of DC [8].

## 6. Oral Manifestations

The most common oral changes in DC patients were oral leukoplakia (80% of the entire DC population), decreased root/crown ratio (75% with sufficient tooth development), and mild taurodontism (57% with sufficient tooth development). Multiple permanent teeth with decreased root/crown ratios further suggest DC. Patients also may have an increased prevalence and severity of periodontal disease [3].

The primary findings of dermal pigmentation, nail dystrophy, and oral leukoplakia are observed in both cases reported here.

## 7. Conclusion

Dentists should take care not to overlook DC when they come across leukoplakia in a young individual with no history of tobacco usage. Proper history taking, clinical examination, relevant blood investigations, and biopsy will suffice to diagnose the condition [2]. Prompt referral to the clinician is essential as early treatment and constant monitoring can greatly increase the life expectancy of the patient [7].
